# (Di-*tert*-butyl­phosphan­yl)bis­(diphenyl­phosphan­yl)phosphane

**DOI:** 10.1107/S1600536808019077

**Published:** 2008-06-28

**Authors:** Aleksandra Wisniewska, Katarzyna Baranowska, Eberhard Matern, Jerzy Pikies

**Affiliations:** a Department of Inorganic Chemistry, Gdańsk University of Technology, 11/12 G. Narutowicz St. 80952-PL Gdańsk, Poland; b Institute for Inorganic Chemistry, University of Karlsruhe, 15 G. Engesserstrasse St., 76131 Karlsruhe, Germany

## Abstract

The title phosphane, C_32_H_38_P_4_ or (Ph_2_P)_2_P(P^*t*^Bu_2_), has a P atom that is linked to another three P atoms in a pyramidal configuration; the P—P distances in the range 2.2231 (7)–2.2446 (7) Å indicate that the P—P bonds are single bonds.

## Related literature

For the synthesis of silylated triphosphanes, see: Kovacs *et al.* (1996 ▶). For other similar pyramidal *iso*tetraphosphanes, see: Cowley *et al.* (1997 ▶); Fritz *et al.* (1987 ▶); Jones *et al.* (2002 ▶). For planar (^*t*^Bu_2_P)_3_P, see: Fritz *et al.* (1999 ▶). For evaluation of NMR data, see: Bruker (1999 ▶); Hägele *et al.* (1987 ▶).
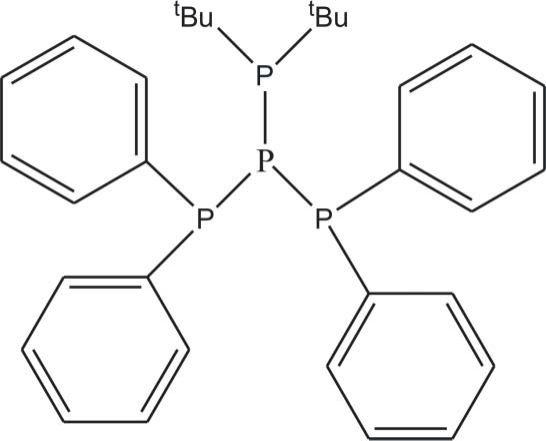


## Experimental

### 

#### Crystal data

C_32_H_38_P_4_
*M* *_r_* = 546.5
Triclinic, 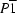

*a* = 10.0161 (6) Å
*b* = 11.9258 (7) Å
*c* = 12.9951 (7) Å
α = 104.831 (5)°
β = 100.201 (5)°
γ = 90.900 (5)°
*V* = 1473.79 (15) Å^3^
*Z* = 2
Mo *K*α radiation
μ = 0.28 mm^−1^
*T* = 120 (2) K
0.32 × 0.15 × 0.13 mm

#### Data collection

Oxford Diffraction KM-4-CCD diffractometer
Absorption correction: none
10351 measured reflections
5474 independent reflections
4356 reflections with *I* > 2σ(*I*)
*R*_int_ = 0.023

#### Refinement

*R*[*F* ^2^ > 2σ(*F* ^2^)] = 0.038
*wR*(*F* ^2^) = 0.106
*S* = 1.07
5474 reflections
331 parameters
H-atom parameters constrained
Δρ_max_ = 0.45 e Å^−3^
Δρ_min_ = −0.30 e Å^−3^

### 

Data collection: *CrysAlis CCD* (Oxford Diffraction, 2006 ▶); cell refinement: *CrysAlis RED* (Oxford Diffraction, 2006 ▶); data reduction: *CrysAlis RED*; program(s) used to solve structure: *SHELXS97* (Sheldrick, 2008 ▶); program(s) used to refine structure: *SHELXL97* (Sheldrick, 2008 ▶); molecular graphics: *ORTEP-3 for Windows* (Farrugia, 1997 ▶); software used to prepare material for publication: *WinGX* (Farrugia, 1999 ▶).

## Supplementary Material

Crystal structure: contains datablocks global, I. DOI: 10.1107/S1600536808019077/ng2467sup1.cif

Structure factors: contains datablocks I. DOI: 10.1107/S1600536808019077/ng2467Isup2.hkl

Additional supplementary materials:  crystallographic information; 3D view; checkCIF report

## Figures and Tables

**Fig. 1.**
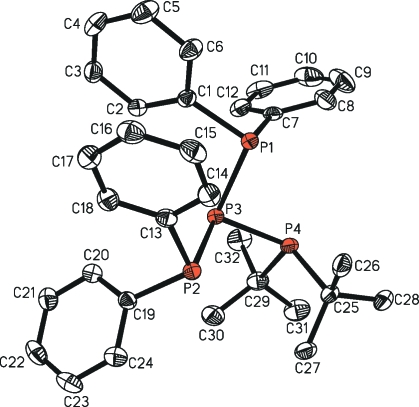
A view of the title molecule, showing the atom-labelling scheme. Displacement ellipsoids are drawn at the 50% probability level and H atoms have been omitted for clarity.

**Fig. 2.**
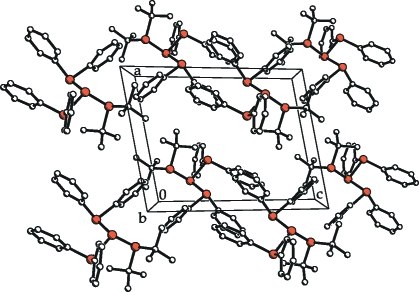
Crystal packing of the title compound, viewed approximately along the b axis.

## supplementary crystallographic information

### Comment

The reaction of Ph_2_PCl with *^t^*Bu_2_P–P(SiMe_3_)Li 1.5 THF in toluene at -30 °C (Kovacs *et al. *1996) yielded unexpectedly (Ph_2_P)_2_P(P*^t^*Bu_2_) (1) in the place of expected Ph_2_P–P(SiMe_3_)–P*^t^*Bu_2_.

The molecular structure of (1) is shown in Fig.1. The geometry around P3 atom in (1) is pyramidal, the sum of angles around P3 is 310.26 degrees. The geometry around P1 atom indicates more pyramidal character (the sum of angles is 297.49 degrees) than around P3. The geometry around P4 atom is more planar (the sum of angles is 320.58 degrees) than around P3. The P–P distances (2.2327 Å - mean value) clearly indicate a single bond character of these bonds. The tendency of phosphane to planarity is more visible for compounds with big groups attached to the central P atom. This assumption is strongly supported by the planar geometry around the central P atom in (*^t^*Bu_2_P)_3_P. This planarity is accompanied by a significant shortening of the P–P distances (2.198 Å) (Fritz *et al. *1987)*.*

### Experimental

A solution of *^t^*Bu_2_P–P(SiMe_3_)Li 1.5 THF (855 mg 3.42 mmol) in 20 ml toluene at -30 °C was dropped to a solution of Ph_2_PCl (792 mg, 3.59 mmol) in 20 ml toluene. The resulting solution was stirred for 3 h at–30 °C and for 12 h at ambient temp. Then the solvent was removed under vacuum at 1 mTorr for 1 h, the residue dissolved in pentane (40 ml), filtered and concentrated to about 8 ml. After 6 days at -35 *^o^*C the solution yielded 688 mg of colourless crystals of (Ph_2_P)_2_P(P*^t^*Bu_2_) (1).

^31^P{^1^H} NMR of (Ph_2_P1,2)_2_P3(P4*^t^*Bu_2_) (1) (Bruker Av400, C_6_D_6_, 298 K, external standard 85% H_3_PO_4_)(δ p.p.m.) 37.9 dt, P4; -19.5 dd*, P1, P2; -62.3 dt*, P3. ^1^J(P3—P4) =–410.7 Hz, ^1^J(P1—P3) = -253.3 Hz, ^2^J(P1—P4) = +33.9 Hz (*= multiplet of higher order).

Chemical shifts and coupling constants of (1) were optimized using Bruke software (Bruker 1999, Hägele *et al. *1987).

### Refinement

All H atoms were refined as riding on C atoms with aromatic C—H = 0.95 Å, methyl C—H = 0.98 Å, and *U*_iso_(H) = 1.2*U*_eq_(C) for CH groups, 1.5*U*_eq_(C) for CH_3_ groups.

### Crystal data

**Table:**

C_32_H_38_P_4_	*Z* = 2
*M**_r_* = 546.5	*F*_000_ = 580
Triclinic, *P*1	*D*_x_ = 1.232 Mg m^−^^3^
Hall symbol: -P 1	Mo *K*α radiation λ = 0.71073 Å
*a* = 10.0161 (6) Å	Cell parameters from 6775 reflections
*b* = 11.9258 (7) Å	θ = 2.4–32.4º
*c* = 12.9951 (7) Å	µ = 0.28 mm^−^^1^
α = 104.831 (5)º	*T* = 120 (2) K
β = 100.201 (5)º	Prism, colourless
γ = 90.900 (5)º	0.32 × 0.15 × 0.13 mm
*V* = 1473.79 (15) Å^3^	

### Data collection

**Table:**

Oxford DiffractionKM-4-CCD diffractometer	4356 reflections with *I* > 2σ(*I*)
Monochromator: graphite	*R*_int_ = 0.023
Detector resolution: 8.1883 pixels mm^-1^	θ_max_ = 25.5º
*T* = 120(2) K	θ_min_ = 2.4º
0.75° wide ω scans	*h* = −11→12
Absorption correction: none	*k* = −14→13
10351 measured reflections	*l* = −15→14
5474 independent reflections	

### Refinement

**Table:**

Refinement on *F*^2^	Secondary atom site location: difference Fourier map
Least-squares matrix: full	Hydrogen site location: inferred from neighbouring sites
*R*[*F*^2^ > 2σ(*F*^2^)] = 0.038	H-atom parameters constrained
*wR*(*F*^2^) = 0.106	*w* = 1/[σ^2^(*F*_o_^2^) + (0.0689*P*)^2^] where *P* = (*F*_o_^2^ + 2*F*_c_^2^)/3
*S* = 1.07	(Δ/σ)_max_ < 0.001
5474 reflections	Δρ_max_ = 0.45 e Å^−^^3^
331 parameters	Δρ_min_ = −0.30 e Å^−^^3^
Primary atom site location: structure-invariant direct methods	Extinction correction: none

### Special details

**Table:**

Geometry. All e.s.d.'s (except the e.s.d. in the dihedral angle between two l.s. planes) are estimated using the full covariance matrix. The cell e.s.d.'s are taken into account individually in the estimation of e.s.d.'s in distances, angles and torsion angles; correlations between e.s.d.'s in cell parameters are only used when they are defined by crystal symmetry. An approximate (isotropic) treatment of cell e.s.d.'s is used for estimating e.s.d.'s involving l.s. planes.
Refinement. Refinement of *F*^2^ against ALL reflections. The weighted *R*-factor *wR* and goodness of fit *S* are based on *F*^2^, conventional *R*-factors *R* are based on *F*, with *F* set to zero for negative *F*^2^. The threshold expression of *F*^2^ > σ(*F*^2^) is used only for calculating *R*-factors(gt) *etc*. and is not relevant to the choice of reflections for refinement. *R*-factors based on *F*^2^ are statistically about twice as large as those based on *F*, and *R*- factors based on ALL data will be even larger.

### Fractional atomic coordinates and isotropic or equivalent isotropic displacement parameters (Å^2^)

**Table:**

	*x*	*y*	*z*	*U*_iso_*/*U*_eq_	
C1	−0.05525 (19)	0.27686 (16)	0.36738 (15)	0.0197 (4)	
C2	−0.10603 (19)	0.16151 (17)	0.33015 (16)	0.0223 (4)	
H2	−0.0706	0.11	0.2741	0.027*	
C3	−0.2075 (2)	0.12062 (18)	0.37356 (17)	0.0267 (5)	
H3	−0.2412	0.0416	0.347	0.032*	
C4	−0.2601 (2)	0.19420 (19)	0.45549 (17)	0.0299 (5)	
H4	−0.3292	0.1659	0.4857	0.036*	
C5	−0.2118 (2)	0.3082 (2)	0.49266 (18)	0.0354 (5)	
H5	−0.2479	0.3592	0.5486	0.042*	
C6	−0.1106 (2)	0.34949 (18)	0.44923 (17)	0.0288 (5)	
H6	−0.0783	0.4288	0.4757	0.035*	
C7	−0.03069 (19)	0.37273 (17)	0.19424 (16)	0.0222 (4)	
C8	−0.0180 (2)	0.48242 (19)	0.17833 (18)	0.0325 (5)	
H8	0.0439	0.5402	0.2289	0.039*	
C9	−0.0951 (3)	0.5082 (2)	0.0890 (2)	0.0446 (7)	
H9	−0.0871	0.5841	0.0792	0.054*	
C10	−0.1834 (3)	0.4244 (2)	0.0143 (2)	0.0431 (6)	
H10	−0.2353	0.4423	−0.0474	0.052*	
C11	−0.1966 (2)	0.3146 (2)	0.02890 (18)	0.0368 (6)	
H11	−0.2575	0.2569	−0.0227	0.044*	
C12	−0.1212 (2)	0.28863 (19)	0.11857 (17)	0.0278 (5)	
H12	−0.1309	0.2131	0.1288	0.033*	
C13	0.24915 (19)	0.19694 (17)	0.47598 (16)	0.0214 (4)	
C14	0.2700 (2)	0.31269 (17)	0.53800 (16)	0.0258 (5)	
H14	0.3222	0.3667	0.5163	0.031*	
C15	0.2149 (2)	0.34903 (18)	0.63082 (18)	0.0297 (5)	
H15	0.2286	0.428	0.6719	0.036*	
C16	0.1403 (2)	0.27079 (19)	0.66379 (17)	0.0313 (5)	
H16	0.1035	0.2958	0.7278	0.038*	
C17	0.1193 (2)	0.15651 (19)	0.60370 (18)	0.0313 (5)	
H17	0.0675	0.103	0.6263	0.038*	
C18	0.1735 (2)	0.11927 (18)	0.51040 (17)	0.0260 (5)	
H18	0.1588	0.0402	0.4697	0.031*	
C19	0.3207 (2)	−0.00176 (16)	0.32067 (15)	0.0206 (4)	
C20	0.2027 (2)	−0.07357 (17)	0.27282 (16)	0.0253 (4)	
H20	0.1186	−0.0399	0.2577	0.03*	
C21	0.2071 (2)	−0.19302 (18)	0.24733 (17)	0.0291 (5)	
H21	0.126	−0.2409	0.2153	0.035*	
C22	0.3292 (2)	−0.24324 (19)	0.26830 (17)	0.0317 (5)	
H22	0.3317	−0.3255	0.251	0.038*	
C23	0.4468 (2)	−0.17404 (19)	0.31416 (18)	0.0334 (5)	
H23	0.5308	−0.2084	0.3279	0.04*	
C24	0.4427 (2)	−0.05407 (18)	0.34036 (16)	0.0269 (5)	
H24	0.5242	−0.0068	0.3722	0.032*	
C25	0.4464 (2)	0.34098 (17)	0.19073 (17)	0.0241 (4)	
C26	0.4359 (2)	0.42053 (18)	0.30219 (18)	0.0300 (5)	
H26A	0.5207	0.469	0.3328	0.045*	
H26B	0.4204	0.3729	0.3508	0.045*	
H26C	0.36	0.4703	0.2941	0.045*	
C27	0.5567 (2)	0.25572 (19)	0.20422 (18)	0.0280 (5)	
H27A	0.5694	0.2103	0.1327	0.042*	
H27B	0.529	0.2032	0.2447	0.042*	
H27C	0.6423	0.299	0.2439	0.042*	
C28	0.4878 (2)	0.41895 (19)	0.1227 (2)	0.0334 (5)	
H28A	0.4212	0.4777	0.1184	0.05*	
H28B	0.4908	0.3714	0.0496	0.05*	
H28C	0.5778	0.4575	0.1567	0.05*	
C29	0.2723 (2)	0.14372 (18)	0.00756 (16)	0.0277 (5)	
C30	0.3239 (2)	0.03535 (18)	0.04003 (18)	0.0333 (5)	
H30A	0.267	0.014	0.087	0.05*	
H30B	0.4181	0.0515	0.0791	0.05*	
H30C	0.3196	−0.0289	−0.0251	0.05*	
C31	0.3551 (2)	0.1739 (2)	−0.07156 (18)	0.0383 (6)	
H31A	0.3423	0.1103	−0.138	0.058*	
H31B	0.4516	0.1854	−0.0379	0.058*	
H31C	0.3242	0.2455	−0.0893	0.058*	
C32	0.1237 (2)	0.1177 (2)	−0.05120 (19)	0.0367 (5)	
H32A	0.1174	0.0502	−0.1139	0.055*	
H32B	0.091	0.1852	−0.0757	0.055*	
H32C	0.0678	0.1009	−0.0015	0.055*	
P1	0.07579 (5)	0.34576 (4)	0.31567 (4)	0.01952 (13)	
P2	0.33268 (5)	0.15796 (4)	0.35823 (4)	0.02017 (14)	
P3	0.17163 (5)	0.19161 (4)	0.22775 (4)	0.01832 (13)	
P4	0.26732 (5)	0.27752 (4)	0.12351 (4)	0.02045 (14)	

### Atomic displacement parameters (Å^2^)

**Table:**

	*U*^11^	*U*^22^	*U*^33^	*U*^12^	*U*^13^	*U*^23^
C1	0.0156 (9)	0.0236 (10)	0.0209 (10)	0.0015 (8)	0.0024 (8)	0.0079 (8)
C2	0.0207 (10)	0.0228 (10)	0.0249 (10)	0.0040 (8)	0.0074 (8)	0.0069 (8)
C3	0.0237 (11)	0.0252 (11)	0.0326 (12)	−0.0018 (9)	0.0030 (9)	0.0117 (9)
C4	0.0218 (11)	0.0421 (13)	0.0278 (11)	−0.0058 (10)	0.0057 (9)	0.0126 (10)
C5	0.0308 (12)	0.0417 (13)	0.0296 (12)	−0.0043 (10)	0.0124 (10)	−0.0021 (10)
C6	0.0249 (11)	0.0296 (12)	0.0279 (11)	−0.0054 (9)	0.0081 (9)	−0.0011 (9)
C7	0.0197 (10)	0.0263 (11)	0.0252 (11)	0.0094 (8)	0.0118 (8)	0.0093 (8)
C8	0.0415 (13)	0.0272 (12)	0.0341 (12)	0.0122 (10)	0.0153 (10)	0.0115 (10)
C9	0.0699 (19)	0.0379 (14)	0.0377 (14)	0.0269 (13)	0.0232 (14)	0.0204 (11)
C10	0.0430 (14)	0.0630 (17)	0.0331 (13)	0.0277 (13)	0.0118 (11)	0.0257 (13)
C11	0.0233 (11)	0.0606 (16)	0.0294 (12)	0.0093 (11)	0.0081 (10)	0.0145 (11)
C12	0.0197 (10)	0.0381 (12)	0.0294 (11)	0.0035 (9)	0.0071 (9)	0.0140 (9)
C13	0.0172 (9)	0.0241 (10)	0.0224 (10)	0.0036 (8)	0.0008 (8)	0.0068 (8)
C14	0.0235 (10)	0.0244 (11)	0.0268 (11)	−0.0003 (9)	−0.0014 (9)	0.0062 (9)
C15	0.0304 (11)	0.0241 (11)	0.0299 (11)	0.0056 (9)	0.0012 (9)	0.0013 (9)
C16	0.0343 (12)	0.0359 (13)	0.0251 (11)	0.0107 (10)	0.0097 (10)	0.0072 (9)
C17	0.0331 (12)	0.0313 (12)	0.0337 (12)	0.0045 (10)	0.0128 (10)	0.0115 (10)
C18	0.0285 (11)	0.0234 (10)	0.0255 (11)	0.0018 (9)	0.0046 (9)	0.0057 (8)
C19	0.0219 (10)	0.0215 (10)	0.0206 (10)	0.0048 (8)	0.0062 (8)	0.0080 (8)
C20	0.0234 (10)	0.0248 (11)	0.0294 (11)	0.0037 (8)	0.0063 (9)	0.0087 (9)
C21	0.0339 (12)	0.0244 (11)	0.0293 (11)	−0.0002 (9)	0.0068 (10)	0.0072 (9)
C22	0.0490 (14)	0.0218 (11)	0.0259 (11)	0.0116 (10)	0.0095 (10)	0.0070 (9)
C23	0.0366 (13)	0.0332 (12)	0.0315 (12)	0.0180 (10)	0.0062 (10)	0.0100 (10)
C24	0.0245 (11)	0.0298 (11)	0.0260 (11)	0.0054 (9)	0.0039 (9)	0.0070 (9)
C25	0.0176 (10)	0.0260 (11)	0.0313 (11)	0.0001 (8)	0.0060 (8)	0.0112 (9)
C26	0.0274 (11)	0.0248 (11)	0.0360 (12)	−0.0062 (9)	0.0071 (10)	0.0046 (9)
C27	0.0171 (10)	0.0351 (12)	0.0346 (12)	0.0035 (9)	0.0054 (9)	0.0138 (9)
C28	0.0248 (11)	0.0353 (13)	0.0473 (14)	0.0004 (9)	0.0126 (10)	0.0196 (11)
C29	0.0271 (11)	0.0330 (12)	0.0227 (11)	0.0047 (9)	0.0073 (9)	0.0048 (9)
C30	0.0362 (13)	0.0275 (11)	0.0338 (12)	0.0067 (10)	0.0133 (10)	−0.0010 (9)
C31	0.0382 (13)	0.0515 (15)	0.0263 (12)	0.0066 (11)	0.0125 (10)	0.0074 (10)
C32	0.0332 (12)	0.0413 (14)	0.0287 (12)	−0.0010 (11)	0.0000 (10)	0.0009 (10)
P1	0.0169 (3)	0.0182 (3)	0.0239 (3)	0.00124 (19)	0.0052 (2)	0.0054 (2)
P2	0.0165 (3)	0.0201 (3)	0.0244 (3)	0.0010 (2)	0.0029 (2)	0.0074 (2)
P3	0.0153 (2)	0.0183 (3)	0.0219 (3)	0.00182 (19)	0.0037 (2)	0.0060 (2)
P4	0.0165 (3)	0.0232 (3)	0.0239 (3)	0.0039 (2)	0.0057 (2)	0.0088 (2)

### Geometric parameters (Å, °)

**Table:**

C1—C2	1.391 (3)	C20—C21	1.381 (3)
C1—C6	1.393 (3)	C20—H20	0.95
C1—P1	1.847 (2)	C21—C22	1.385 (3)
C2—C3	1.385 (3)	C21—H21	0.95
C2—H2	0.95	C22—C23	1.376 (3)
C3—C4	1.384 (3)	C22—H22	0.95
C3—H3	0.95	C23—C24	1.386 (3)
C4—C5	1.369 (3)	C23—H23	0.95
C4—H4	0.95	C24—H24	0.95
C5—C6	1.385 (3)	C25—C27	1.530 (3)
C5—H5	0.95	C25—C26	1.537 (3)
C6—H6	0.95	C25—C28	1.535 (3)
C7—C8	1.383 (3)	C25—P4	1.902 (2)
C7—C12	1.395 (3)	C26—H26A	0.98
C7—P1	1.845 (2)	C26—H26B	0.98
C8—C9	1.382 (3)	C26—H26C	0.98
C8—H8	0.95	C27—H27A	0.98
C9—C10	1.376 (4)	C27—H27B	0.98
C9—H9	0.95	C27—H27C	0.98
C10—C11	1.378 (4)	C28—H28A	0.98
C10—H10	0.95	C28—H28B	0.98
C11—C12	1.380 (3)	C28—H28C	0.98
C11—H11	0.95	C29—C30	1.529 (3)
C12—H12	0.95	C29—C31	1.535 (3)
C13—C18	1.394 (3)	C29—C32	1.535 (3)
C13—C14	1.400 (3)	C29—P4	1.902 (2)
C13—P2	1.833 (2)	C30—H30A	0.98
C14—C15	1.387 (3)	C30—H30B	0.98
C14—H14	0.95	C30—H30C	0.98
C15—C16	1.382 (3)	C31—H31A	0.98
C15—H15	0.95	C31—H31B	0.98
C16—C17	1.378 (3)	C31—H31C	0.98
C16—H16	0.95	C32—H32A	0.98
C17—C18	1.388 (3)	C32—H32B	0.98
C17—H17	0.95	C32—H32C	0.98
C18—H18	0.95	P1—P3	2.2305 (7)
C19—C24	1.394 (3)	P2—P3	2.2446 (7)
C19—C20	1.397 (3)	P3—P4	2.2231 (7)
C19—P2	1.838 (2)		
			
C2—C1—C6	117.64 (18)	C22—C23—C24	119.9 (2)
C2—C1—P1	126.30 (15)	C22—C23—H23	120
C6—C1—P1	116.03 (15)	C24—C23—H23	120
C3—C2—C1	120.96 (18)	C23—C24—C19	121.0 (2)
C3—C2—H2	119.5	C23—C24—H24	119.5
C1—C2—H2	119.5	C19—C24—H24	119.5
C2—C3—C4	120.36 (19)	C27—C25—C26	109.64 (17)
C2—C3—H3	119.8	C27—C25—C28	108.98 (17)
C4—C3—H3	119.8	C26—C25—C28	107.24 (17)
C5—C4—C3	119.4 (2)	C27—C25—P4	117.57 (14)
C5—C4—H4	120.3	C26—C25—P4	106.54 (13)
C3—C4—H4	120.3	C28—C25—P4	106.38 (14)
C4—C5—C6	120.4 (2)	C25—C26—H26A	109.5
C4—C5—H5	119.8	C25—C26—H26B	109.5
C6—C5—H5	119.8	H26A—C26—H26B	109.5
C5—C6—C1	121.2 (2)	C25—C26—H26C	109.5
C5—C6—H6	119.4	H26A—C26—H26C	109.5
C1—C6—H6	119.4	H26B—C26—H26C	109.5
C8—C7—C12	119.11 (19)	C25—C27—H27A	109.5
C8—C7—P1	117.40 (16)	C25—C27—H27B	109.5
C12—C7—P1	123.49 (15)	H27A—C27—H27B	109.5
C9—C8—C7	120.2 (2)	C25—C27—H27C	109.5
C9—C8—H8	119.9	H27A—C27—H27C	109.5
C7—C8—H8	119.9	H27B—C27—H27C	109.5
C10—C9—C8	120.3 (2)	C25—C28—H28A	109.5
C10—C9—H9	119.8	C25—C28—H28B	109.5
C8—C9—H9	119.8	H28A—C28—H28B	109.5
C9—C10—C11	120.1 (2)	C25—C28—H28C	109.5
C9—C10—H10	120	H28A—C28—H28C	109.5
C11—C10—H10	120	H28B—C28—H28C	109.5
C10—C11—C12	120.0 (2)	C30—C29—C31	110.04 (18)
C10—C11—H11	120	C30—C29—C32	108.77 (18)
C12—C11—H11	120	C31—C29—C32	107.76 (18)
C11—C12—C7	120.3 (2)	C30—C29—P4	115.98 (14)
C11—C12—H12	119.8	C31—C29—P4	109.73 (15)
C7—C12—H12	119.8	C32—C29—P4	104.12 (14)
C18—C13—C14	118.53 (19)	C29—C30—H30A	109.5
C18—C13—P2	124.94 (15)	C29—C30—H30B	109.5
C14—C13—P2	116.45 (15)	H30A—C30—H30B	109.5
C15—C14—C13	120.47 (19)	C29—C30—H30C	109.5
C15—C14—H14	119.8	H30A—C30—H30C	109.5
C13—C14—H14	119.8	H30B—C30—H30C	109.5
C16—C15—C14	120.2 (2)	C29—C31—H31A	109.5
C16—C15—H15	119.9	C29—C31—H31B	109.5
C14—C15—H15	119.9	H31A—C31—H31B	109.5
C17—C16—C15	120.0 (2)	C29—C31—H31C	109.5
C17—C16—H16	120	H31A—C31—H31C	109.5
C15—C16—H16	120	H31B—C31—H31C	109.5
C16—C17—C18	120.3 (2)	C29—C32—H32A	109.5
C16—C17—H17	119.8	C29—C32—H32B	109.5
C18—C17—H17	119.8	H32A—C32—H32B	109.5
C17—C18—C13	120.51 (19)	C29—C32—H32C	109.5
C17—C18—H18	119.7	H32A—C32—H32C	109.5
C13—C18—H18	119.7	H32B—C32—H32C	109.5
C24—C19—C20	118.16 (18)	C7—P1—C1	99.44 (9)
C24—C19—P2	115.67 (15)	C7—P1—P3	96.19 (6)
C20—C19—P2	126.16 (15)	C1—P1—P3	101.86 (6)
C21—C20—C19	120.70 (19)	C13—P2—C19	103.58 (9)
C21—C20—H20	119.7	C13—P2—P3	101.37 (6)
C19—C20—H20	119.7	C19—P2—P3	100.37 (6)
C20—C21—C22	120.2 (2)	P4—P3—P1	97.92 (3)
C20—C21—H21	119.9	P4—P3—P2	109.51 (3)
C22—C21—H21	119.9	P1—P3—P2	102.93 (3)
C23—C22—C21	119.98 (19)	C25—P4—C29	110.55 (9)
C23—C22—H22	120	C25—P4—P3	112.04 (7)
C21—C22—H22	120	C29—P4—P3	97.98 (7)
